# Immunolocalization of p53 and p21 in Kidneys Exposed to T-2 Mycotoxin

**DOI:** 10.3390/cimb47121045

**Published:** 2025-12-15

**Authors:** Piret Hussar, Katerina Blagoevska, Monika Dovenska, Lazo Pendovski, Florina Popovska-Percinic

**Affiliations:** 1 Faculty of Medicine, University of Tartu, 50411 Tartu, Estonia; 2 Institute of Veterinary Medicine and Animal Sciences, Estonian University of Life Sciences, 51006 Tartu, Estonia; 3 Faculty of Veterinary Medicine, Ss. Cyril & Methodius University in Skopje, 1000 Skopje, North Macedonia; katerinab@fvm.ukim.edu.mk (K.B.); mdovenska@fvm.ukim.edu.mk (M.D.); lpendovski@fvm.ukim.edu.mk (L.P.); florinap@fvm.ukim.edu.mk (F.P.-P.)

**Keywords:** T-2 mycotoxicosis, p53, p21, immunohistochemistry, kidneys

## Abstract

T-2 mycotoxins are known to induce toxic effects in animals. The kidneys are particularly vulnerable to oxidative stress induced by toxins, resulting in cellular damage, apoptosis, and disruptions to cell cycle regulation. Cyclin-dependent kinase inhibitor p21 and tumor suppressor protein p53 are key modulators of these pathways. As our knowledge on the immunolocalization of p53 and p21 during T-2 mycotoxicosis in the avian kidney is limited, this study was designed to investigate the immunolocalization of these two critical apoptosis regulatory proteins in the renal tissues of broiler chickens treated with T-2 mycotoxin. In the experiment, ten seven-day-old female Ross chickens (*Gallus gallus domesticus*) were separated into the control group and T-2 toxin group. T-2 toxin was orally administered to the T-2 toxin group for three days. Then, 24 h after the last dose, chickens were sacrificed and kidney tissues were collected and fixed for immunohistochemical staining. Immunohistochemical analysis using polyclonal primary antibodies against p53 and p21 (Abcam, Cambridge, UK) demonstrated increased expression of p21 and p53 in T-2 toxin-treated chickens’ kidneys compared to healthy chickens in the control group. Both proteins were mainly localized in the epithelial cells of the renal proximal tubules. The enhanced staining intensity of p21 and p53 emphasizes their contribution to T-2-induced renal toxicity and suggests their potential as biomarkers for the early detection of nephrotoxicity.

## 1. Introduction

T-2 mycotoxin, produced by the genus *Fusarium*, is known for its toxic effects in various organs and organ systems, presenting considerable risks to both animals and humans, while also resulting in significant economic losses for the cereal industry [1,2]. The primary sources of trichothecenes are tainted maize, wheat, oats, rye and barley [3]. Mycotoxins are widespread in animal feed [4]. A recent survey of 23,003 feed samples collected across China between 2021 and 2024 revealed significant contamination, with 9.7%, 2.7%, and 15.7% of raw feed ingredients exceeding the safety limits for Aflatoxin B1 (AFB1), Deoxynivalenol (DON), and Zearalenone (ZEN), respectively. Furthermore, the co-occurrence of two or more mycotoxins was frequently observed in all analyzed samples. Specifically, the co-contamination rate of Aflatoxin B1, Deoxynivalenol, and Zearalenone ranged from 60% to 100% in over 70% of the feed samples. These findings highlight the urgent need for ongoing monitoring and research to develop effective strategies for preventing and mitigating mycotoxin co-contamination. Over 20 aflatoxins were identified, with Aflatoxin B1 (AFB1) being the most toxic [5]. To date, key glutathione S-transferase (GST) isozymes—GSTA2X, GSTA3, GSTT1L, GSTZ1-1, and GSTZ1-2—have been identified in chickens as playing crucial roles in detoxifying AFB1, suggesting potential nutritional or genetic strategies to alleviate aflatoxicosis.

T-2 toxin is considered the most toxic trichothecene among mycotoxins [3,6]. Changes in metabolic pathways have been described in organs of the immune and gastrointestinal systems, such as the thymus, spleen, liver, stomach, and duodenum [7,8,9,10]. Besides attacking the reproductive organs in mature organisms, it has been demonstrated that T-2 toxin can readily cross the placental barrier in pregnant mammals, leading to toxicity in the developing embryo [11].

After the administration of T-2 mycotoxin, damage occurs in all vital organs—the heart, brain, lungs, liver, and kidneys. In the cardiovascular system, cardiac toxicity induced by T-2 toxin is characterized by increased capillary permeability, hemorrhages, necrosis, the infiltration of inflammatory cells, and cardiomyocyte death in the heart [12]. T-2 toxin exposure has been shown to induce cardiac fibrosis and dysfunction. Cardiac injury caused by trichothecene mycotoxin exposure is characterized by mitochondrial dysfunction, reactive oxygen species (ROS), endoplasmic reticulum stress, inflammatory responses, autophagy, the peroxisome proliferator-activated receptor-gamma (PPAR-γ) signaling pathway, and the transforming growth factor-beta 1 (TGF-β1)/Smad2/3 signaling pathway.

In brain tissue, mycotoxins are able to penetrate the neurovascular barrier, causing cellular oxidative stress and neuroinflammatory response, which leads to cellular damage and apoptosis in neuronal cells [13]. In neurotoxic pathways, aflatoxin B1 (AFB1) and T-2 mycotoxin induce neural injury in the hippocampus and cerebral cortex, resulting in impaired synaptic signaling and ultimately disrupting overall nervous system function. The pathophysiological process in brain tissue is associated with elevated reactive oxygen species (ROS), redox imbalance, amplified inflammatory response, mitochondrial impairment, autophagic process, and cellular apoptosis. 

In the respiratory system, T-2 mycotoxin causes nasal pruritus, discomfort, sneezing, and rhinorrhea; bronchopulmonary and tracheobronchial toxicity can lead to breathlessness, stridor, and coughing, as well as blood-tinged sputum [14]. The toxin inhibits protein biosynthesis by interacting with the ribosome, impairs mitochondrial activity, activates mitogen-activated protein kinases (MAPKs), induces the infiltration of alveolar macrophages and neutrophils, stimulates cytokine release, causes pulmonary hemorrhage, and damages tissues. Studies have shown that the inhalation of a toxic dose of mycotoxin results in systemic effects without causing lung injury. However, when mycotoxins are ingested, they can lead to chronic inflammation in the lungs. Mycotoxins can also induce chronic obstructive pulmonary disease in farm animals.

Histopathological analysis has shown that T-2 mycotoxin causes pathological alterations in liver tissue, including hepatocyte edema, increased cellular volume, and an increased number of cytoplasmic granules, indicating that exposure to T-2 induces hepatocyte apoptosis [15]. At the molecular level, the apoptosis mediated by T-2 through the mitochondria was driven by ROS production and the translocation of cytochrome c between the mitochondria and cytoplasm.

After kidney tissues are exposed to T-2 mycotoxin, the degeneration of mitochondria, apoptosis and necrosis of the renal proximal and distal tubules, karyomegaly, and the binucleation of epithelial cells have been noted [6]. The pathohistological findings show alterations that depend on both dose and duration. Investigations in juvenile goats after 30 days of exposure to a T-2 toxin-contaminated diet demonstrated the degeneration of renal tissue, including damage to the epithelial lining of the proximal and distal convoluted tubules, renal tubular necrosis, mitochondrial and nuclear degeneration with heterochromatin condensation, and a disrupted nuclear membrane [16]. The affected epithelial cells exhibited a loss of cristae, resulting in the formation of empty spaces and the transformation of mitochondria into pleomorphic forms, with variable sizes and shapes, including rounded, dumbbell, and curved structures. Biochemical analysis revealed elevated blood urea nitrogen and serum creatinine levels. Additionally, a significant increase in oxidative stress markers, such as malondialdehyde, along with a decrease in superoxide dismutase, catalase, and glutathione levels in the kidneys, underscored the enhanced role of free radicals in kidney damage. The primary histological alterations in the kidneys included widespread vacuolar degeneration and hypertrophy of the tubular epithelial cells. In studies on Wistar rats, following 12 weeks on a toxin-contaminated diet, nearly all animals showed signs of severe degeneration in the epithelial cells in their proximal convoluted tubules, leading to lumen obstruction, with denuded cells and proteinaceous material present within the lumina [17]. Karyomegaly and binucleation were also observed in epithelial cells, as well as mononuclear cell infiltration around the glomeruli and within the interstitial compartment. In a study investigating the effects of varying mycotoxin doses on apoptosis occurrence in swine renal epithelial cells, trichothecene T-2 toxin was administered at concentrations of 2.5 µM and 25 µM [18]. T2 toxin at a concentration of 2.5 µM caused apoptosis in 6.9% of cells, whereas at a concentration of 25 µM, it caused apoptosis in 26.35% of cells.

It is known that, in the kidneys, toxins cause tubular necrosis and intracellular stress, triggering various response mechanisms [19,20,21,22]. These stressors often result in DNA damage, which in turn activates critical elements of the damage response pathway, including the tumor suppressor protein p53 and the cyclin-dependent kinase (CDK) inhibitor p21. In response to DNA damage, p53 serves as a key regulator of cell cycle arrest, apoptosis, and senescence; if the damage is irreparable, p53 can trigger apoptosis or, alternatively, inhibit cell cycle progression by transactivating p21 [23,24,25]. Thus, while p53 and p21 normally protect the organism by halting the division of damaged cells and preventing the spread of mutations, an imbalance in their activity can lead to detrimental consequences [26,27,28]. Insufficient activity allows damaged cells to continue dividing, increasing the risk of cancer, while excessive activity may promote cellular senescence or apoptosis, resulting in tissue degeneration, impaired regeneration, and accelerated aging. Consequently, the precise regulation of p53 and p21 is essential in maintaining cellular and tissue homeostasis.

Although the impacts of T-2 mycotoxin are well documented, and it is established that mycotoxin T-2 causes apoptosis, the molecular pathways involved remain unclear [18,29]. To date, research has focused on the function of p53 and p21 in the DNA damage response, the regulation of the cell cycle, and apoptosis; however, specific studies investigating the immunolocalization and expression of these proteins in kidney tissue are less common. Experiments investigating their expression in the context of kidney damage are more common within scientific studies dealing with a variety of toxins and chemicals, while work on mycotoxin T-2 and its effects in kidney tissue has focused more on macroscopic and histological changes. As our knowledge on the immunolocalization of p53 and p21 during T-2 mycotoxin-induced kidney damage in the avian kidney is limited, despite studies on the effects of T-2 mycotoxins on different organs on a molecular level pointing toward the p53-dependent apoptosis pathway, the aim of this study is to investigate the immunolocalization of the two critical apoptosis-regulatory proteins—p53 and p21—in the renal tissue of broiler chickens subjected to T-2 mycotoxin exposure. Understanding the expression patterns of these proteins can provide insights into the molecular mechanisms underlying renal toxicity.

## 2. Materials and Methods

In this study, ten layer-type 7-day-old Ross chickens (*Gallus gallus domesticus*) were obtained from a commercial Macedonian hatchery. The chickens were divided equally into control (healthy chicken, n = 5) and T-2 toxin groups (n = 5). Chickens were raised in temperature-controlled brooders under standard conditions, with a day–night cycle of 23 h:1 h. Feed and water were provided ad libitum. In the T-2 toxin group, T-2 mycotoxin (Sigma, Darmstadt, Germany; Cat. No. T4887) was dissolved in water and administrated orally (0.250 mg/chick/day) for three consecutive days, beginning on the fourth day after hatching. Twenty-four hours after the administration of the final toxin dose, chickens were euthanized via an intracardiac overdose of 0.5 mL of 20% sodium pentobarbital. Kidney specimens of 0.5–1.0 cm in diameter were collected, fixed in 10% neutral buffered formalin, dehydrated through a series of alcohol solutions, and embedded in paraffin. Nine 7 μm thick slices from each chicken’s renal tissue were prepared (six slices for further division and staining with two antibodies and three slices for negative controls) using a Leica 2135 microtome (Leica microsystems, Wetzlar, Germany). Subsequently, the tissue sections were deparaffinized using xylene and rehydrated through a graded ethanol series. For immunohistochemical staining (IHC), polyclonal primary antibodies p21 ((ab18209) Anti-p21 antibody, Abcam, Cambridge, UK) and p53 ((ab1431) Anti-p53 (phospho S15) antibody, Abcam, Cambridge, UK) were applied, along with the IHC kit containing the corresponding secondary antibodies, following the manufacturer’s guidelines (IHC kit, Abcam, Cambrige, UK). After blocking endogenous peroxidase activity with 3% H_2_O_2_, the sections were subjected to heat-mediated antigen retrieval in sodium citrate buffer (pH 6) for 20 min and then incubated with primary antibodies at 1:1000 dilution for 30 min at 37 °C. Biotinylated secondary antibody and streptavidin-conjugated peroxidase, both included in the IHC kit, were used for detection, with DAB as the chromogen. Nuclei were counterstained with Harris Hematoxylin. Negative controls for T-2 mycotoxin-treated chicken kidneys consisted of antibody diluent (Dako, S0809, Glostrup, Denmark) instead of primary antibodies. Positive controls to identify p21 and p53 were obtained from the antibody manufacturer’s website (https://www.abcam.com/en-us/products/primary-antibodies/p21-antibody; https://www.abcam.com/en-us/products/primary-antibodies/p53-antibody; accessed on 1 January 2023), where examples of antibody reactions in kidney tissues are provided.

The IHC study was repeated twice to verify the results of the initial trial. The immunolocalization of p21 and p53 was assessed by two researchers using visual inspection in a blinded analysis. The intensity of protein expression in the chicken renal tubules was categorized as weak (+), moderate (++), or strong (+++). Images of the slides were captured using a PreciPoint M8 digital microscope (PreciPoint, Munich, Germany). Statistical analysis was performed with Python software (version 3.12.4, 2024). A paired *t*-test was conducted to compare the control and T-2 toxin groups.

The experimental protocol was approved by the Ethical Committee of Ss. Cyril and Methodius University in Skopje, in accordance with the guidelines outlined in the European Convention for the Protection of Vertebrate Animals used for Experimental and Other Scientific Purposes (ETS no. 123, Appendix A) (No. 03-7534 from 12.04.2013).

## 3. Results

### 3.1. Immunohistochemistry

**Figure 1:**
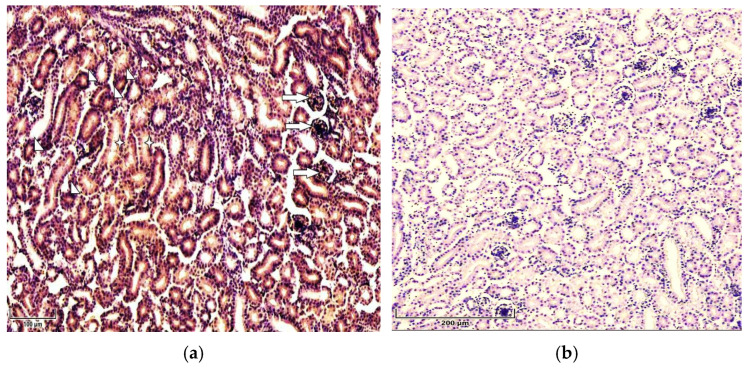
Immunolocalization of p53 in 7-day-old chickens: (**a**) Strong expression of p53 is seen in renal proximal (arrowheads) and distal (asterisks) tubules and in glomeruli (arrows) in chicken kidneys in T-2 toxin group (n = 5). Scale bar: 100 µm. (**b**) Weak expression of p53 is seen in renal tissue of control group chickens (n = 5). Scale bar: 200 µm.

Immunohistochemical analysis demonstrated the immunolocalization of p21 and p53 in the renal tissue of broiler chickens. Strong expression of both studied proteins, p53 and p21, was observed in the epithelial cells of the proximal convoluted tubule (PCT) in chickens exposed to T-2 toxin. The two proteins were predominantly localized in the nuclei of the epithelial cells of the PCT; however, a moderate staining of the cytoplasm was observed. Besides the strong expression of p53 in PCT, it was immunolocalized in glomerular cells and, to a lesser extent, in the cells of the distal tubules, as well as in the endotheliocytes of small blood vessels in the birds from the T-2 toxin group (Figure 1a).

**Figure 2:**
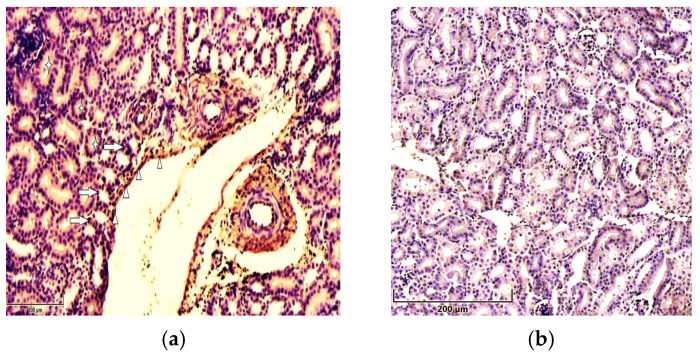
Immunolocalization of p21 in 7-day-old chickens: (**a**) the cytoplasm (asterisks) and nuclei (arrows) of renal tubular epithelial cells and vessels’ endothelial cells (arrowheads) show strong staining for p21 in the kidneys of chickens in the T-2 toxin group (n = 5). Scale bar: 100 µm. (**b**) Weak expression of p51 in the renal tissues of chickens in the control group (n = 5). Scale bar: 200 µm.

Compared to the control group, intense expression of p21 was observed in the nuclei and cytoplasm of the epithelial cells of the PCT, as well as in the nuclei of endothelial cells in the sinusoids of chickens in the T-2 toxin group (Figure 2a). Additionally, the expression of p21 was observed in the endothelial cells of small blood vessels and DCTs and, to a lesser degree, in glomerular cells. No significant expression of either of the antibodies studied, p53 or p21, was noted in the kidneys of chickens in the control group (Figure 1b and Figure 2b.

**Figure 3:**
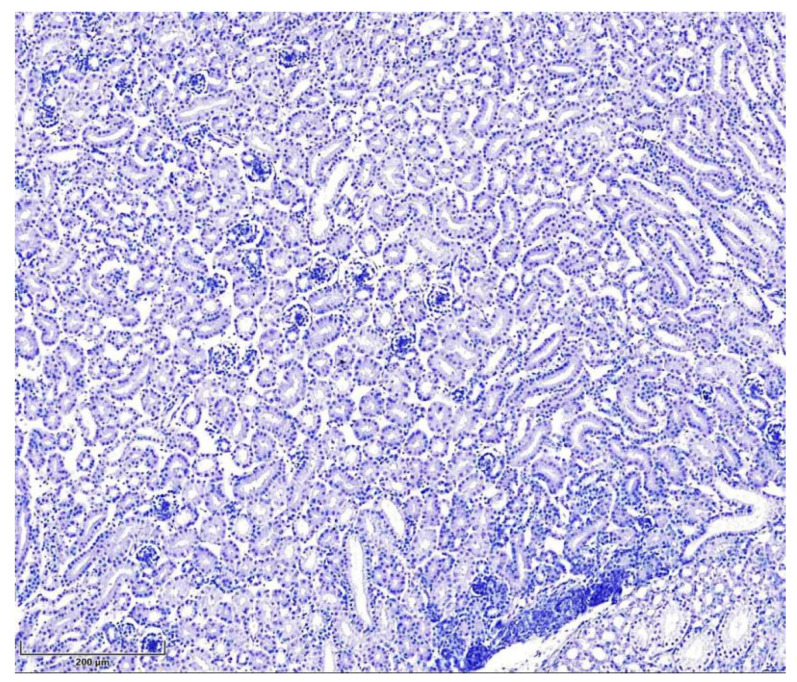
Seven-day-old intoxicated chicken kidney tissue, negative control (without primary antibodies, n = 5). Hematoxylin, scale bar: 200 µm.

The negative control, T-2-mycotoxicated chicken kidneys containing antibody diluent instead of primary antibodies, showed no specific staining (Figure 3).

### 3.2. Statistical Analysis

The statistical analysis was conducted using Python software (version 3.12.4, 2024), which allows for the conversion of qualitative data obtained from the IHC study into quantitative form. Numerical values (Table 1) were assigned to the staining intensity—+ = 1 (weak), ++ = 2 (moderate), and +++ = 3 (strong)—and the statistical calculations were performed for both groups. During the analysis, the mean staining intensity for the study groups was determined, along with the variability (standard deviation = SD) within each group (Table 2). Both proteins showed a significantly higher mean intensity in the T-2 mycotoxin group compared to the control. A paired *t*-test revealed a statistically significant difference (*p* < 0.05) for both proteins, confirming that these differences were significant with *p*-values less than 0.05. These results indicate that exposure to T-2 mycotoxin significantly increases the expression of both the proteins studied.

## 4. Discussion

Secondary metabolites known as mycotoxins are produced by several molds, including *Penicillium*, *Aspergillus*, *Stachybotrys*, and *Fusarium* spp. [18]. Posing a considerable hazard to both animal and human health, mycotoxins can penetrate the organism not only via ingestion but also through the inhalation of contaminated air. A wide spectrum of toxic effects has been documented, including nephrotoxicity, hepatotoxicity, immunotoxicity, dermotoxicity, and estrogenic and carcinogenic actions, as well as mutagenic and teratogenic properties and adverse impacts on the hematopoietic system [3]. Mycotoxins such as trichothecenes, aflatoxins, fumonisins, zearalenone, and ochratoxin A are capable of inducing apoptosis. Apoptosis, programmed cell death, can be initiated through one of two pathways: intrinsic or extrinsic [30]. In the intrinsic pathway, also referred to as the mitochondrial pathway, cells respond to internal stress signals by triggering self-destruction. The extrinsic pathway, alternatively known as the tumor necrosis factor pathway, is activated by extracellular signals originating from other cells. Programmed cell death represents a physiological process that occurs during both embryogenesis and the postnatal period. Aberrations during its course can result in the development of a range of pathological conditions [31].

**Figure 4:**
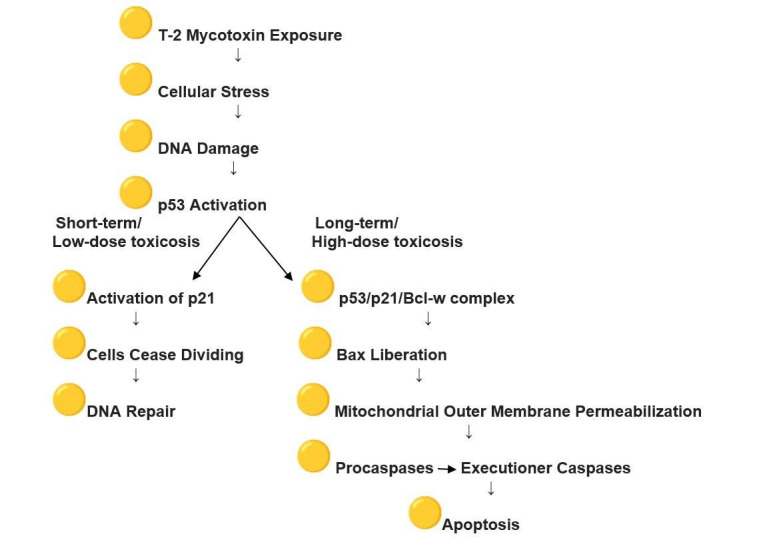
Effect of T-2 mycotoxin exposure on p53 and p21 activation.

Studies on kidney tissue after T-2 mycotoxin exposure have revealed apoptotic changes in the kidneys’ tubular epithelium, causing tubular necrosis and intracellular stress, which trigger various response mechanisms, including the p53-mediated apoptosis pathway [32]. In response to genotoxic or cellular stress, the expression of numerous genes involved in senescence, growth arrest, or apoptosis is regulated by p53, also known as tumor protein p53, cellular tumor antigen p53 (UniProt name), or transformation-related protein 53 (TRP53), a regulatory transcription factor [33,34]. The p53 proteins are crucial in vertebrates, as they prevent cancer formation [35]. The p53-mediated apoptosis pathway is one of the main apoptosis signaling pathways, with the p53 protein stimulating both the intrinsic and extrinsic versions [36]. Apoptosis is ultimately triggered by a cascade of caspase activation, including Caspase-8 and Caspase-3, initiated through the formation of the Death-Inducing Signaling Complex and the engagement of specific “death” receptors belonging to the Tumor Necrosis Factor Receptor family, as part of the extrinsic pathway. The release of Cytochrome-C from the mitochondria is primarily regulated by the Bcl-2 protein family, which dominates the intrinsic apoptotic pathway. This family includes both anti-apoptotic (pro-survival) and pro-apoptotic members. In the intrinsic apoptosis signaling cascade, mitochondrial outer membrane permeabilization is induced by p53 through its interactions with the multidomain members of the Bcl-2 family [37]. p53, a tumor suppressor, interacts with pro-survival Bcl-2 family proteins such as Bcl-w and Bcl-X_L,_ thereby releasing Bax, which subsequently exerts pro-apoptotic or anti-invasive functions, depending on the cellular stress context. Kim et al. (2017) showed that, even though p53 is capable of binding Bcl-w independently, the suppression of cell invasion and induction of cell death require the liberation of Bax, which depends on p21 [38]. By forming a p53/p21/Bcl-w complex, p21 bound to Bcl-w in a manner that preserved all pairwise interactions between p53/p21, p21/Bcl-w, and p53/Bcl-w, thereby allowing Bax to be released from the complex. Overall, the findings indicated that the regulation of cell invasion and apoptosis is mediated primarily by the p53/p21 complex rather than by p53 alone through its targeting of Bcl-2 family proteins. Belonging to the CIP/Kip family of CDK inhibitors, cyclin-dependent kinase (CDK) inhibitor p21—also referred to as p21^waf1/cip1 or P21/CDKN1A—is a small regulatory protein [39]. CDK p21, a cyclin-dependent kinase inhibitor, participates in cell cycle arrest during G1/S transition, a p53-dependent process in which p53 serves as the principal transcriptional regulator of p21. In cells, the expression of p21 can be induced by the persistent activation of p53, resulting in the cessation of cell division and leading to senescence, in which cells are no longer capable of proliferation. Functioning primarily as a tumor suppressor, p21 inhibits cell cycle progression and facilitates DNA repair. In the absence of cellular stress, p53 and p21 levels are kept low within the cell [40,41]. In accordance with data in the literature, our study showed low p53 expression in renal tubular epithelial cells, with minimal localization in the cytoplasm and nuclei of cells from the control group chickens [42]. However, 24 h after exposure to T-2 mycotoxins, a marked enhancement in p53 expression was noted, particularly in the nuclei of many cells in the PCT. Our study observed nuclear accumulation of p53, reflecting a sequence of events initiated by T-2 mycotoxin exposure and cellular stress, leading to DNA damage and subsequent activation of p53, as described in the literature discussed above. An illustrative schematic based on these referenced studies is provided in Figure 4.

In our study, unexposed chickens exhibited weak nuclear expression of p21 in renal tubular epithelial cells. Conversely, after T-2 mycotoxin exposure, p21 exhibited strong nuclear localization, particularly in the proximal tubular epithelial cells. The intense nuclear staining of p21 in these cells proved that p21 was activated as a downstream target of p53, mediating cell cycle arrest and facilitating DNA damage repair mechanisms. Besides nuclear staining, some renal tubular cells exhibited slight cytoplasmic p21 staining, suggesting its participation in apoptotic or senescence pathways. As the current preliminary study on the immunolocalization of p53 and p21 in chicken renal tissue under normal conditions and in T-2 mycotoxicosis was limited by the relatively small number of birds (n = 5), future studies should include larger experimental groups to enhance statistical power. Additionally, complementary approaches such as mRNA quantification or Western blot analysis could provide further insights into cellular responses to mycotoxins.

## 5. Conclusions

The experimental investigation showed that both proteins studied—p21 and p53—are present in higher amounts in the renal tissue of chickens in the mycotoxin-treated group compared to the control group of unexposed chickens. The pronounced nuclear presence of p53 and p21 in the kidney tissue of T-2-exposed broiler chickens indicates the activation of the DNA damage response to cellular stress induced by T-2 exposure. p21 expression was detected within tubular cell nuclei and showed a strong correlation with p53 localization. These results highlight the simultaneous participation of p21 and p53 in renal toxicity induced by T-2 mycotoxin and provide a potential biomarker for the early detection of mycotoxin-induced nephrotoxicity.

## Figures and Tables

**Table 1:** p53 and p21 expression analysis of 7-day-old chicken kidneys.

Antibody	p53 *		p21 *	
Chicken Group **	T-2 Group	Control Group	T-2 Group	Control Group
Proximal convoluted tubules	+++	+	++	+
Distal convoluted tubules	++	+	++	+
Endothelium	++	+	++	+
Glomerulus	++	+	+/++	+

* The staining intensity, assessed visually, is categorized as follows: + weak; ++ moderate; +++ strong. ** The T-2 toxin group and control group each included 5 chickens (n = 5).

**Table 2:** Mean intensity and statistical significance (SD) of p53 and p21 expression in renal tissue of healthy (control group) and T-2 toxin-treated (T-2 group) chickens *.

Protein	Mean Intensity T-2 Toxin	SD T-2 Toxin	Mean Intensity Control Group	SD Control Group	Paired *t*-Test *p*-Value ^1^
p53	2.25	0.50	1.00	0.00	0.0154
p21	1.88	0.25	1.00	0.00	0.0060

* n = 5. ^1^ *p* < 0.05.

## Data Availability

The original contributions presented in this study are included in the article material. Further inquiries can be directed to the corresponding author.

## Abbreviations

The abbreviations listed below are employed in this manuscript.

p53	Tumor protein p53; *TP53*, cellular tumor antigen p53, *TRP53*
p21	Cyclin-dependent kinase inhibitor 1A; *CDKN1A*, p21^WAF1/Cip1^
AFB1	Aflatoxin B1
DON	Deoxynivalenol
ZEN	Zearalenone
GST	Glutathione S-transferase
PPAR-γ	Peroxisome proliferator-activated receptor-gamma
TGF-β1	Transforming growth factor-beta 1
ROS	Reactive oxygen species
MAPK	Mitogen activated protein kinase
CIP/Kip	CDK interacting protein/Kinase inhibitory protein
IHC	Immunohistochemistry
PCT	Proximal convoluted tubules
DCT	Distal convoluted tubules
SD	Standard deviation
TRP53	Transformation-related protein 53
Bcl	B-cell lymphoma
Bcl Xl	B-cell lymphoma-extra large
Bcl-w	BCL-2-like protein 2
Bax	Bcl-2-associated X protein
